# Social Stratification and Cooperative Behavior in Spatial Prisoners' Dilemma Games

**DOI:** 10.1371/journal.pone.0131005

**Published:** 2015-07-14

**Authors:** Peng Lu, Xiaoping Zheng

**Affiliations:** 1 Department of Automation, Tsinghua University, Beijing, China; 2 Department of Sociology, Tsinghua University, Beijing, China; Peking University, CHINA

## Abstract

It has been a long-lasting pursuit to promote cooperation, and this study aims to promote cooperation via the combination of social stratification and the spatial prisoners’ dilemma game. It is previously assumed that agents share the identical payoff matrix, but the stratification or diversity exists and exerts influences in real societies. Thus, two additional classes, elites and scoundrels, derive from and coexist with the existing class, commons. Three classes have different payoff matrices. We construct a model where agents play the prisoners’ dilemma game with neighbors. It indicates that stratification and temptation jointly influence cooperation. Temptation permanently reduces cooperation; elites play a positive role in promoting cooperation while scoundrels undermine it. As the temptation getting larger and larger, elites play a more and more positive and critical role while scoundrels’ negative effect becomes weaker and weaker, and it is more obvious when temptation goes beyond its threshold.

## Introduction

This work aims to promote cooperation via the mechanism of social classification under the paradigm of prisoners’ dilemma game. Deemed as the vital element of human societies [1, 2], cooperation brings public goods, and it therefore has been a long-lasting pursuit for scientists to promote cooperation [3–5].

Plenty of methods have been proposed to promote it [2–6], and mechanism design [7–9] is widely applied in traditional game theories, such as the prisoner’s dilemma game [4, 5, 9, 10] and public goods games [11–20], which commonly take on two forms, network games [6, 8, 9, 12, 20] and spatial games [3–5, 9, 10, 12, 15, 21]. However, the traditional game induces two challenges [22–24]: first, agents are subjective, not repeating actions mechanically and randomly. They have memory [8, 12, 22, 24], trust [2, 7, 8, 10, 11, 25–28], expectation [29], altruism [13,16], volunteerism [19], recommendation [14], prestige and reputation [4, 6, 7, 12, 19, 23, 28–30]. It indicates that cooperation can be promoted based on these subjective factors; second, homogeneity should be denied, and heterogeneity is supposed to be introduced [11, 20, 25], such as group diversity [8, 23, 31], social structure [26], and network [26, 31]. Heterogeneity fits the real world better, and it is found that cooperation can be enhanced if individual heterogeneity and social structure are applied, such as social influence [22], social norms [30], wealth [17], capability [25], popularity [21], personal values [32], and so on.

This study follows the second orientation. Unlike previous work that merely investigates one type of agents, three classes are introduced. The concept of social class and stratification is heavily discussed in social sciences, in terms of social stratification [33, 34], social structure [35, 36], social cliques [37], social blocs [38], and so on. In real situations, classes and stratification do exist and agents belong to different classes or cliques. Although there have been examples of agents’ classification [11,12], the concept of social stratification is scarcely utilized. Thus, the main motivation is to combine social classification and game theory to figure out the effect of stratification and the condition where cooperation can be enhanced, under the spatial prisoners’ dilemma game.

## Materials and Methods

In order to investigate the effect of stratification, the baseline or class model is applied to compare with the class model. There are commons, elites, and scoundrels in the class model and merely commons in the baseline model.

### Social Stratification: One Class to Three Classes

**Fig 1:**
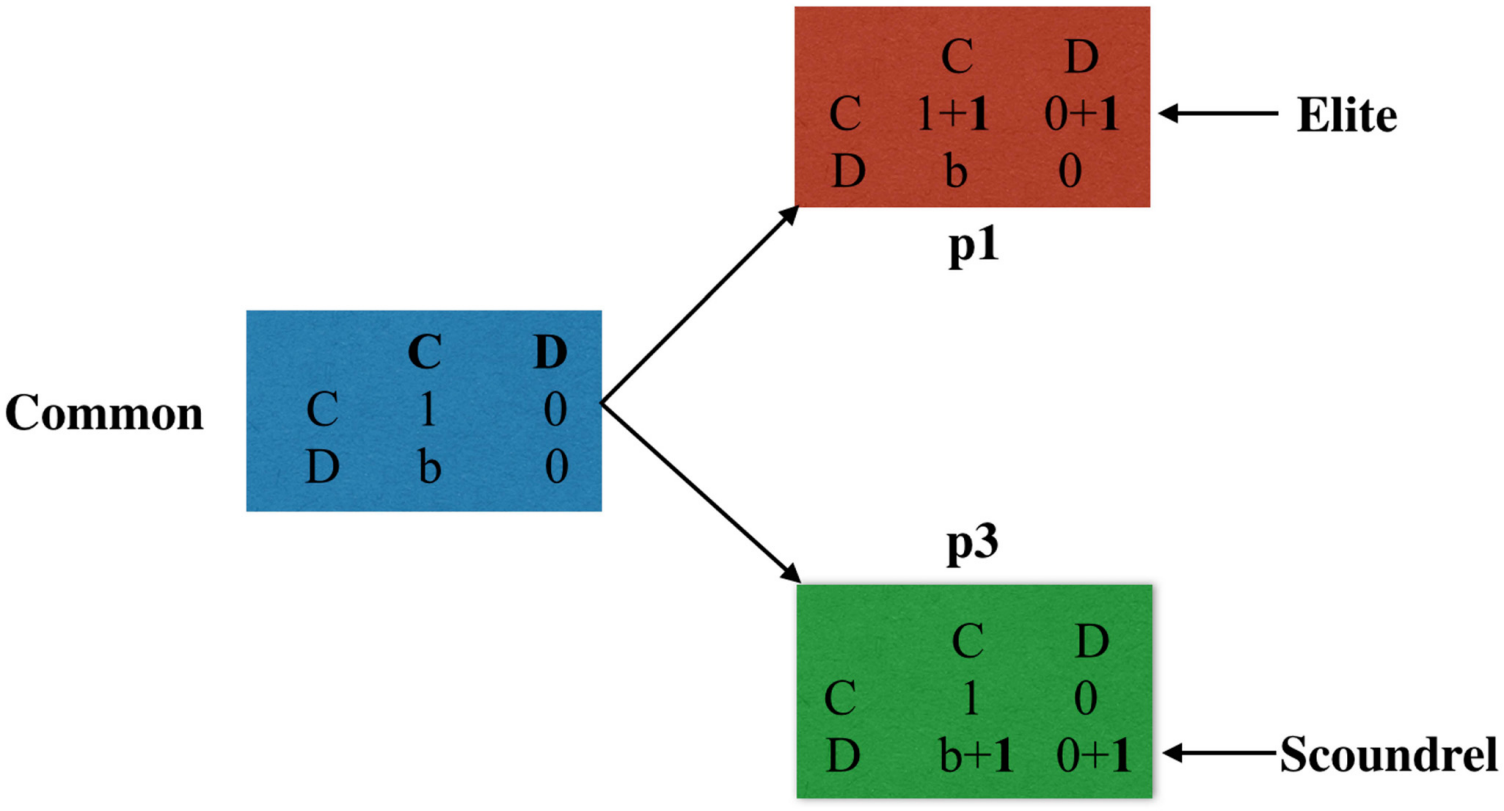
Social Stratification: Common, Elite, and Scoundrel. It illustrates the process of stratification. Two classes, elites and scoundrels derived from the class of commons. The blue block represents the class of commons that has regular payoff matrix. The red block refers to the class of elites that have higher payoffs than the commons when cooperating, i.e. 2 > 1 and 1 > 0. Likewise, the blue block denotes the class of scoundrels that has higher payoffs than the common, i.e. b+1 > 1 and 1 > 0. Elites are highly proud of cooperating and therefore shame of defection, while scoundrels feel shame of cooperating and prone to defect.

Social stratification refers to the process that one class is divided into several classes. Agents are all commons initially, and elites and scoundrels derive from the commons thereafter. The feature of classes is the payoff matrix. For commons, agents who cooperate get payoff 1 with cooperative partners but 0 with defective partners. Meanwhile, people who defect receive payoff b with cooperative partners, and 0 with defective partners. Parameter b satisfies 1 < *b* ≤ 2, and b measures the temptation of defection in that agents that defect on cooperative partners get higher payoffs than they cooperate with their partners. This matrix coincides with previous work [4, 6, 12] and can be seen in Fig 1 as the blue block. The baseline model contains only one class, the commons. In order to investigate the effect of cooperation, we compare the baseline model with the class model.

The class model is derived from the baseline model, and it embraces two new classes, elites and scoundrels, in addition to commons. Some pioneering work has been done in regard to elites [25], which usually exist in the real world. For reasons of responsibility [25], social values [32], and so on, they feel honor and happy of cooperating and receive higher payoffs when they cooperate. Accordingly, the first row of elites is one unit higher than commons in Fig 1. Contrary to elites, scoundrels get higher payoff than average when defecting, which may be caused by social values [32] or preferences [21]. Therefore, the second row of scoundrels is one unit higher than the commons.

### Symmetric and Asymmetric Class Models

**Fig 2:**
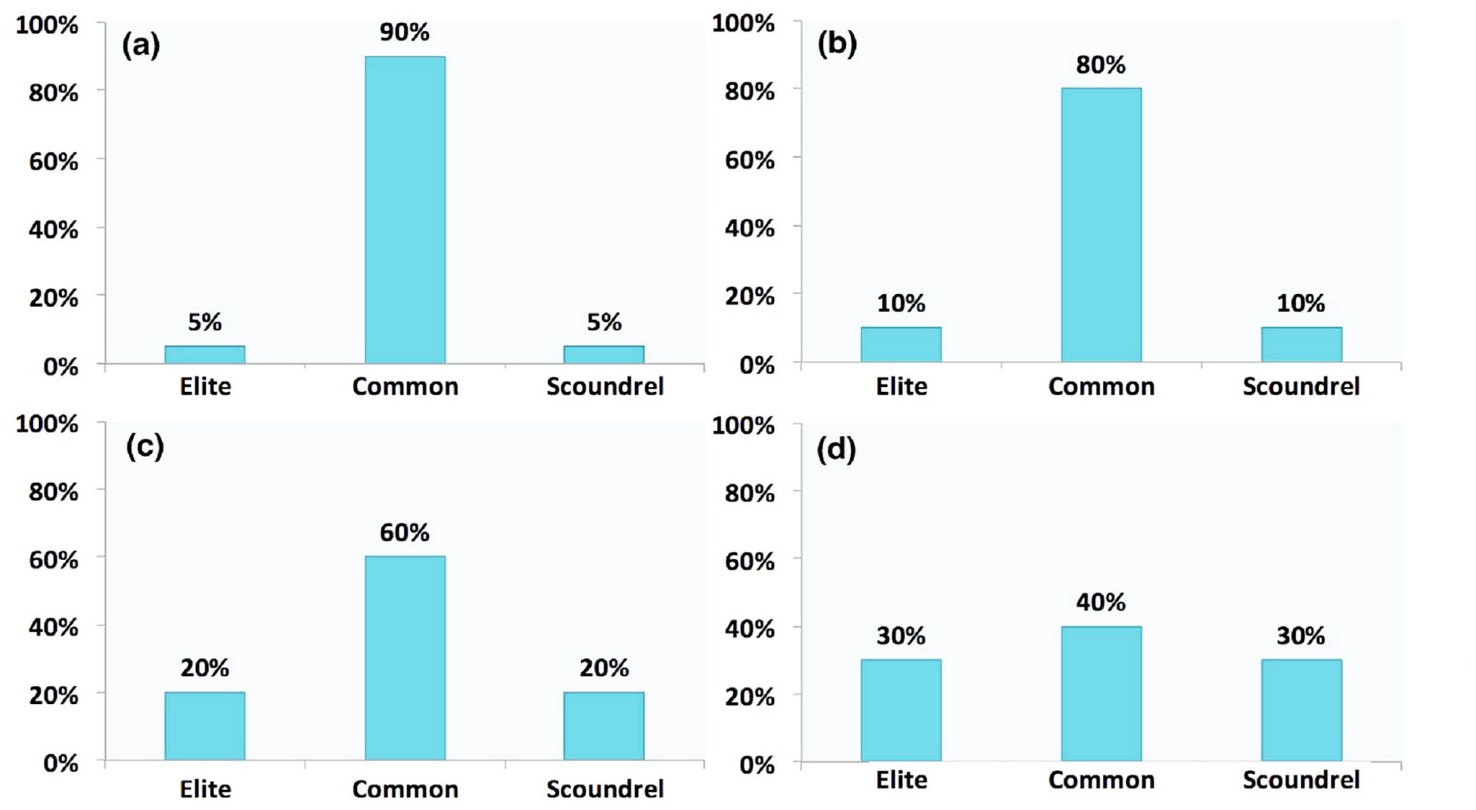
The Symmetric Class Model. Three classes mix together, p1, p2, and p3 denoting percentages of elites, commons, and scoundrels. Symmetric model features p1 = p3, i.e. classes of elites and scoundrels share the same proportions of the population. (a) depicts the symmetric class setting when p1 = p3 = 0.5; (b) depicts the symmetric model when p1 = p3 = 0.1; (c) and (d) depicts the situation of p1 = p3 = 0.2 and 0.3.

**Fig 3:**
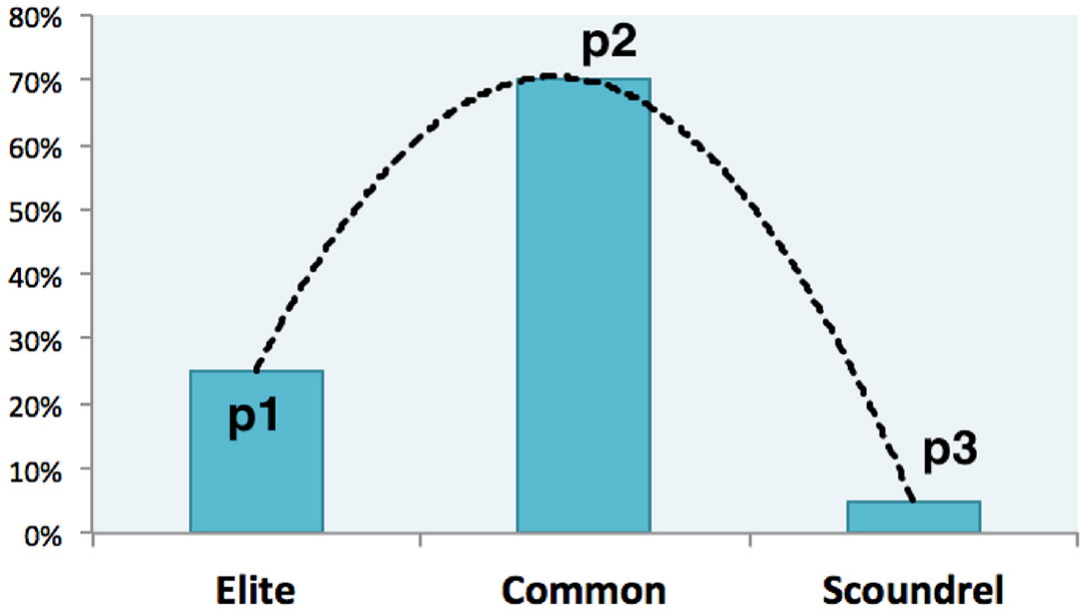
The Asymmetric Class Model. The asymmetric class model allows *p*1 ≠ *p*3 and therefore fits real situations more. these parameters, p1, p2, and p3 freely take values from the unit interval [0, 1]. Symmetric model confounds the effects of elites and scoundrels, mixing them together. Hence, symmetric models can merely identify the effects of temptation and the commons class. Whereas, the asymmetric model makes it possible to investigate pure effects of elites and scoundrels and compare them.

In the class model, the stratification is indicated by a vector (p1, p2, p3), where p1, p2 and p3 denote proportions of elites, commons, and scoundrels respectively. The symmetric class model assumes that two new classes, elites and scoundrels, are symmetrically distributed, which requires that p1 equals p3, while the percentage of the common equals (1-p1-p3). The symmetric class distributions can be seen in Fig 2. The asymmetric class model allows that p1 does not equal p3, which is more flexible and realistic. Fig 3 refers to the asymmetric model, where p1 and p3 take values freely from the unit interval as long as the sum of them is one.

For symmetric class models, the stratification effects measure both effects of elites and scoundrels. Therefore, the symmetric model gives us the overall effect of elites and scoundrels. We apply the symmetric model to figure out how the total cooperation rate evolves with equal numbers of good (elites) and bad agents (scoundrels), as in reality both of these two classes are minorities. The asymmetric model is able to provide pure effects of each class, in that p1 and p3 can be different.

### Strategy Updating

Agents play the prisoners’ dilemma game with eight neighbors on a square lattice. Strategy updating is probabilistic other than deterministic, which means that elites may defect sometimes, and scoundrel might cooperate for a while as well. Strategy updating is determined by the transition probability shown in Eq (1). For each agent, $P_{S_{c}\to S_{a}}$ denotes the transition probability of shifting from a current strategy *S*_*c*_ to an alternative action *S*_a_, and they produce payoff *u*_*c*_ and *u*_*a*_ respectively. If the alternative payoff *u*_*c*_ is larger than the current, the focal agent tends to adopt the alternative strategy, and vise versa [5, 39]. Parameter *β* represents the intensity of selection (*β* → 0 leads to random drift while *β* → ∞ deterministic imitation). As is not the focus of this article, it is assumed that *β* ≡ 1. In this work, *P*_1→0_ denotes the possibility for one who cooperates this time defects next. Likewise, *P*_0→1_ denotes the probability for one who defects cooperates next time, i.e. it denotes the cooperation propensity.

$$P_{S_{c}\to S_{a}}\;=\;\frac{1}{1+\text{exp}\left[(u_{a}-u_{c})\beta \right]}$$ (1)

The number of agents is 4000, i.e. 200*200, and agents of three classes are well mixed on a square lattice. The initial cooperation rate is 50%; the temptation b takes numbers from the set {1.1, 1.2, 1.3, 1.4, 1.5, 1.6, 1.7, 1.8, 1.9, 2.0}; p1 or p3 takes on values from the set {0, 0.025, 0.05, 0.075, 0.1, 0.125, 0.15, 0.175, 0.2, 0.225, 0.25, 0.275, 0.3,0.325,0.35,0.375,0.4}. At each time or iteration, agents of three classes play games with neighbors based on the payoff matrix of their own class in Fig 1. As most of them arrive equilibriums within 20 iterations, the simulation process is conducted for 50 iterations for each combination of parameters. The cooperation rate *ρ*_*c*_, averaged transition probabilities, and other parameters will be recorded and stored at each simulation.

## Outcomes of Symmetric Class

From the perspective of mechanism design [6, 7, 8], we have two mechanisms that influence the cooperation rate of the society: the temptation mechanism seduces good guys such as commons and elites to defect. The temptation influences both individual choices and the cooperation rate; the stratification mechanism allocates elites and scoundrels randomly and renders agents of three classes to play games or interact with each other. Social stratification would be influential to individual choices especially to commons and the cooperation rate as well.

Therefore, the temptation mechanism and stratification mechanism jointly influence individual choices and cooperation levels. In this section, we mainly investigate how temptation and stratification influences individual choices and group cooperation. For each class, we use the averaged transition probability $\overset{-}{P_{0\to 1}}$ to measure the cooperation propensity and investigate how the two mechanisms influences individual choices of three classes and the overall cooperation level.

### Elites’ Cooperation Propensity

**Fig 4:**
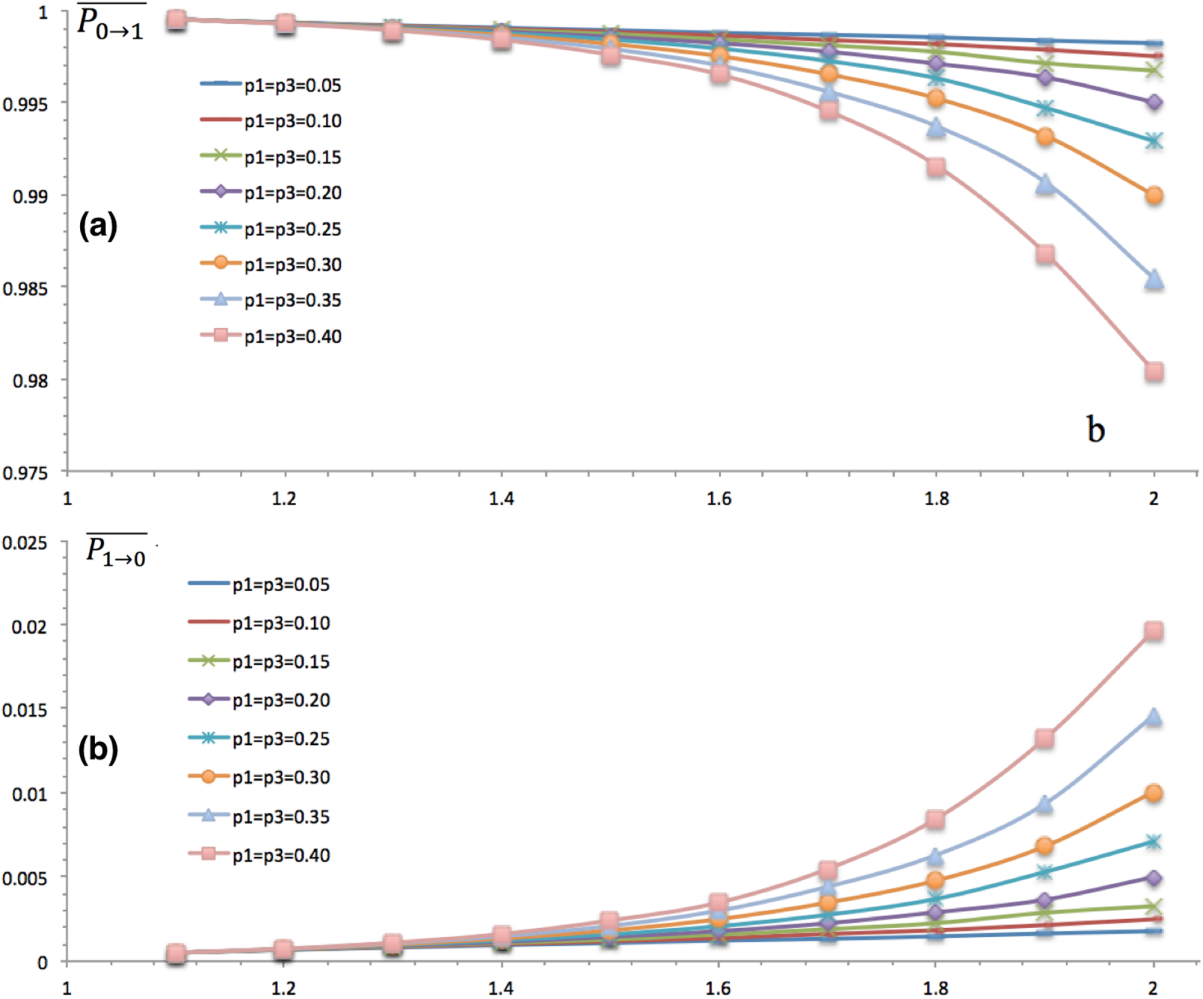
Elites’ Propensities of Cooperation and Defection. The horizontal axis stands for levels of temptation b, and the vertical axis refers to the transition probabilities $\overset{-}{P_{0\to 1}}$ and $\overset{-}{P_{1\to 0}}$, both of which are under the joint influence of the temptation effect and stratification effect. (a) depicts the probability for elites who defect last time cooperate this time. The temptation effect is obvious, in that $\overset{-}{P_{0\to 1}}$ goes down as b increases, which implies that as the temptation is large enough even elites may defect some times. As well, the stratification effect is readily to read that a deeper stratification, i.e. higher p1 and p3, reduces cooperation; (a) and (b) are symmetric to each other, and (b) indicates that both larger temptation and higher stratification lead elites to defect. In all, (a) and (b) indicate that both of temptation and stratification tend to reduce cooperation for elites.

Both the mechanisms of stratification and temptation affect agents’ transition probability, including elites. Elites are highly prone to cooperate, in that their averaged cooperation propensity $\overset{-}{P_{0\to 1}}$ is close to one for all levels of stratification. We have two findings in Fig 4 regarding stratification and temptation: first, stratification reduces cooperation as well. For elites, the more elites and scoundrels there are, the less the cooperation propensity is; second, temptation reduces elites’ cooperation more and more. Although highly cooperative, elites’ averaged propensity to cooperate $\overset{-}{P_{0\to 1}}$ declines as b grows, in that they get larger payoff than cooperate as b grows. The second derivative of cooperation at temptation is negative, which means elites have stronger and stronger propensities to defect. Therefore, both stratification and temptation reduce the cooperation propensity of elites.

### Scoundrels’ Cooperation Propensity

**Fig 5:**
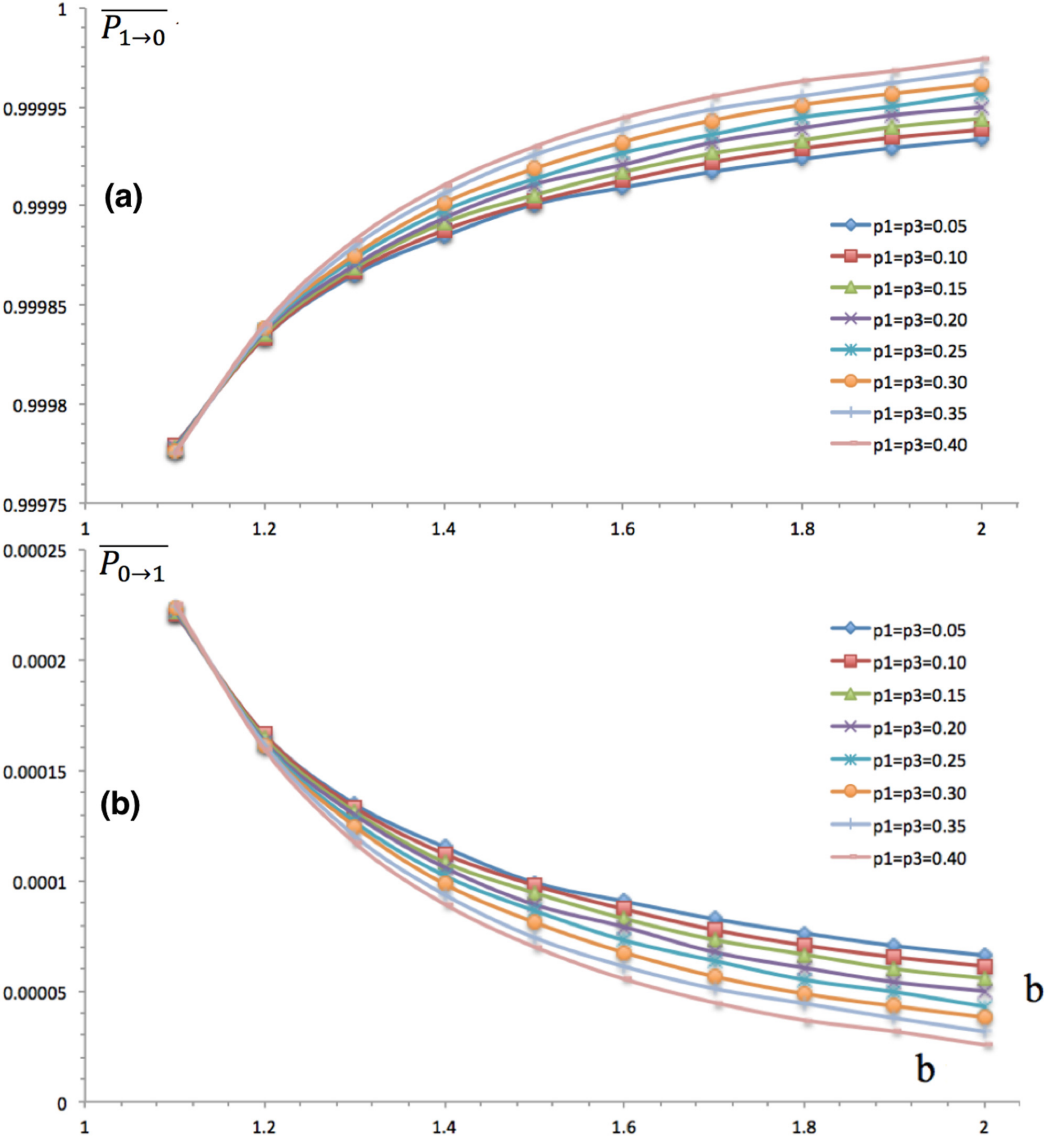
Scoundrels’ Propensities of Cooperation and Defection. The horizontal axis stands for levels of temptation b, and the vertical axis refers to the transition probabilities $\overset{-}{P_{0\to 1}}$ and $\overset{-}{P_{1\to 0}}$ that are under the joint influence of temptation and stratification. (a) depicts the probability for scoundrels who cooperate this time defect for the next, and $\overset{-}{P_{1\to 0}}$ is quite close to 1, which is the core trait of scoundrels. As temptation gets larger and larger, scoundrels are more prone to defect. As p1 and p3 gets larger and larger, scoundrels are more prone to cooperate as well. (b) depicts their joint influence on $\overset{-}{P_{0\to 1}}$. As temptation goes up, scoundrels feel more reluctant to cooperate. As p1 and p3 get bigger and bigger, they are more likely to defect. Both (a) and (b) indicates that temptation and stratification all reduce cooperation.

Scoundrels are naturally prone to defect, which is indicated by Fig 5, where their averaged cooperation rate is close to zero. Although the cooperation rate is low enough, it still decreases as b grows in that temptation permanently reduces cooperation [1, 4, 6], regardless of the class. However, it declines less and less with temptation for scoundrels as the second derivative is positive, which is unlike elites. Besides of the temptation effect, there still exists the stratification effect. As p3 increases, their averaged cooperation level decreases as well, and the defection rate goes up synchronously. Both stratification and temptation play negative roles in promoting the cooperation propensity of scoundrels.

### Commons’ Cooperation Propensity

**Fig 6:**
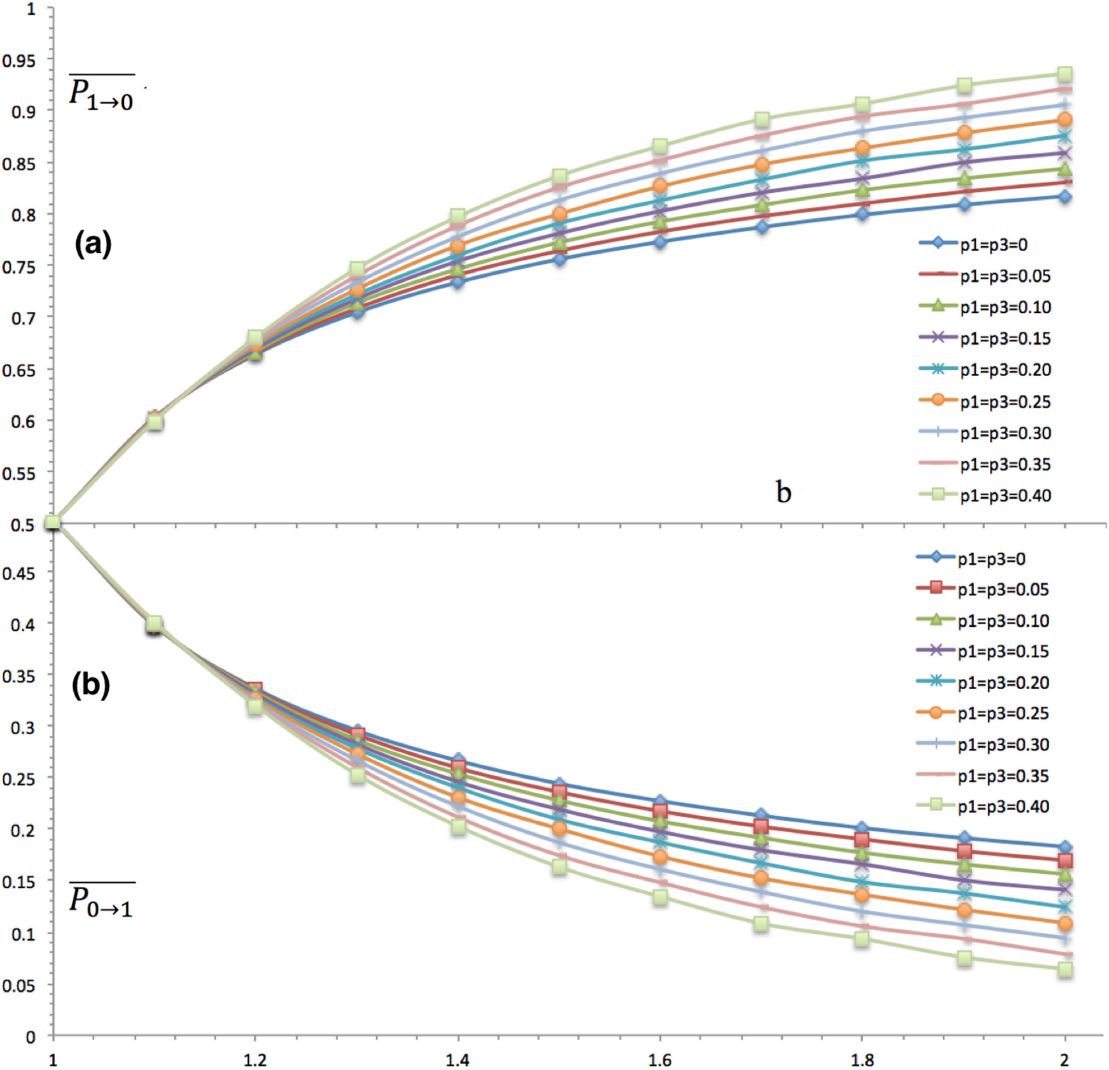
Common’s Propensities of Cooperation and Defection. The commons interact with other commons and the other two classes, elites and scoundrels. The horizontal axis stands for levels of temptation b, and the vertical axis refers to the transition probabilities. It indicates here they are under the joint influence of temptation and stratification. (a) depicts the probability for scoundrels who cooperate this time defect for the next, and $\overset{-}{P_{1\to 0}}$ is close to 0.5, the initial cooperation rate, when b is smaller. However, when temptation grows, it grows gradually towards 1. And as p1 or p3 grows from 0, i.e. no stratification, to 0.40, i.e. strong stratification, they are more prone to defect in the future. (b) depicts the same pattern with (a) that both temptation and stratification undermine cooperation in symmetrical models.

As well, commons’ tendency of cooperation is influenced by these two mechanisms, which is shown by Fig 6. Common’s payoff matrix is the original one [4] and the averaged form of other two classes. When b = 1, there is no difference between defection and cooperation, thus they chose to cooperate or defect randomly and the propensity is therefore 0.5. As b is larger than one and increases gradually, cooperation declines and defect prevails in that defection makes more profit than cooperation. Besides of temptation, stratification affects cooperation as well. As more and more minorities, elites and scoundrels, are present, commons’ cooperation propensity turns to be lower and lower. The main reason is that the probability that a common randomly meets a scoundrel or elite is getting larger and larger as p1 or p3 increases. Scoundrels continually seduce commons to defect while the elites encourages them to cooperate. Under the circumstance that commons encounter same numbers of elites and scoundrels, they would like to defect because the expected payoff of defection is larger than that of cooperation.

### The Overall Cooperation Propensity

**Fig 7:**
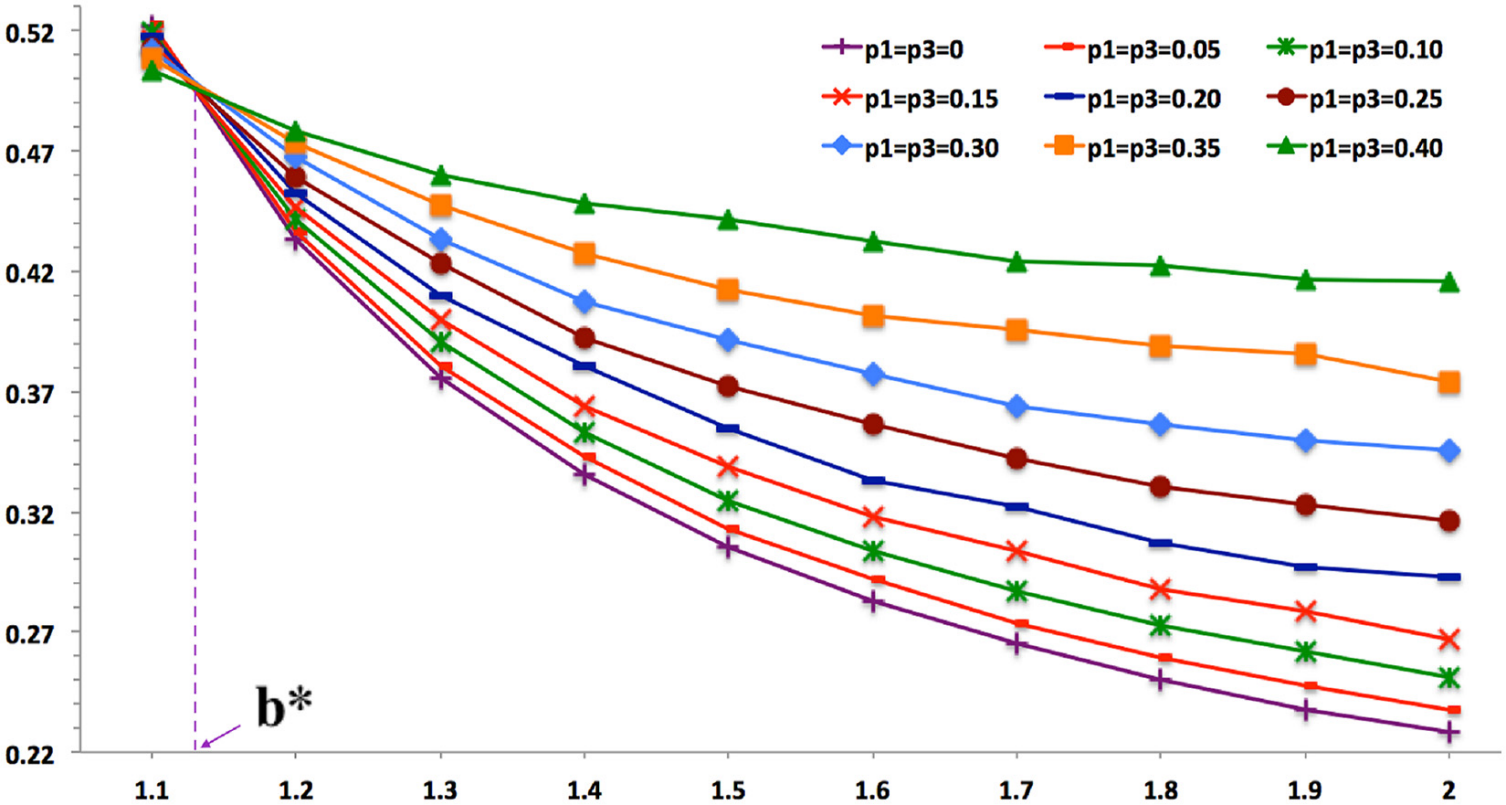
Overall Cooperation Propensity. The x-axis stands for b, and the y-axis denotes the cooperation rate or percentage *ρ*_*C*_. It is obvious that temptation reduces the group cooperation rate, in that for each curve or stratification situation, *ρ*_*C*_ declines with b grows. Unlike temptation, the stratification effect is different based on b. There exists a temptation threshold b*, and stratification reduces cooperation when b < b* and promote it when b < b*.

The averaged cooperation propensity of all agents in last ten iterations is taken to measure the overall cooperation propensity P_0→1_, which is under the joint influence of stratification and temptation as well in Fig 7. As temptation increases, the cooperation propensity decreases, which coincides to previous work [2, 6]. However, stratification shows distinct effects. Stratification defers the effect of temptation, in that curves with more class stratification, i.e. larger p1 and p3, are steeper than those with less stratification. Hence, there is a threshold of temptation b*, above which stratification successfully countervails temptation while below which it does not. In other words, the cooperation rate with more stratification is higher than those with less stratification when b > b*, which is caused by relative forces of stratification and temptation. The stratification mechanism conquers temptation mechanism when b > b*, and it fails to do that when b < b*, which causes the threshold phenomenon of b.

## Outcomes of Asymmetric Class

Besides of symmetric class models, outcomes of asymmetric class model that allows p1 ≠ p3 need to be investigated as well. There are two reasons to do that. First, the asymmetric model fits the reality better as p1 = p3 takes little place while p1 ≠ p3 prevails; Second, the symmetric model confounds or confuses effects of stratification and temptation, which makes it hard to figure out pure effects of them. Based on these considerations, the asymmetric model is applied and related parameters are traversed. As statistical methods are usually applied to analyze outcomes previously [7, 11, 17, 18, 22, 39], pure effects of temptation and stratification can be statistically evaluated.

### Stratification versus Temptation

Results of asymmetric class model indicate that there are two mechanisms influencing cooperation. The asymmetric model makes it possible to compare pure effects of elites, scoundrels, and temptations. A statistical model is applied in Eq (2), where the dependent variable is the overall cooperation rate *ρ*_*C*_, and the independent variables or factors are proportions of elites and scoundrels, and temptation. Parameters, *β*_1_, *β*_1_, and *β*_3_ represent the pure effects on cooperation of elites, scoundrels, and temptations. Besides, C is the constant term and e represents the residual term.

$$\rho_{c}\;=\;C+\beta_{1}∙p1+\beta_{2}∙p2+\beta_{3}∙p3+e$$ (2)

Table 1 gives three effects and each of them is statistically significant. Also, these three factors explain most part of the propensity, which is 92.18%. For temptation, its pure effect is negative (-0.21), which means that temptation always seduces individuals to defect; For elites, its effect is positive (0.51), which indicates that elites will encourage commons to cooperate; For scoundrels, they influence commons to defect other than cooperate in that its coefficient is negative (-0.19).

**Table 1:** Effects of Elites, Scoundrels, and Temptation.

Factors	Coefficients ^a^
β_1_	.5133(.0089)*** ^b^
β_3_	-.1949(.0089)***
β_2_	-.2099(.0035)***
Constant	.6317(.0063)***

Table 1 shows the outcome of OLS regression for Eq (2), where the p1, p2, and p3 jointly affect ρ_c_, with β_1_, β_2_, and β_3_ as their coefficients or effects. These coefficients are all statistically significant at the 0.001 level.

^a^. Significance Level:

*** p < 0.001 (two-tailed).

^b^. The model fit well, and the adjusted R^2^ is 0.9218.

### Elites versus Scoundrels

As temptation permanently reduces cooperation [1, 2, 4], it becomes a priority to find the condition where the effect of elites overcomes that of scoundrels at same levels of temptation, i.e.*β*_1_(·|*b*) > *β*_3_(·|*b*). If elites conquer scoundrels, the cooperation of society will be promoted. In order to figure out relative effects of elites and scoundrels on *ρ*_*C*_, temptation is controlled, and Eq (2) is statistically evaluated at different levels of b. Thus, related coefficients would vary with b. As it assumes that there are no other factors influencing *ρ*_*C*_, we set no constant item in order to compare pure effects of elites and scoundrels.

$$\rho_{c}\left(∙|b\right)\;=\;\beta_{1}(∙|b)∙p1+\beta_{3}(∙|b)∙p3+e$$ (3)

Table 2 gives the outcome of statistical evaluation, and evaluated coefficients fit well because they jointly explain over 97% of the total variance, which is close to 100%. Table 2 indicates that for all levels of b, the elite plays a positive role in enhancing cooperation, while the scoundrel undermines it. Hence, cooperation will be enhanced and well maintained if the whole society embraces more elites. And cooperation will be undermined or defection will prevail as long as there are more scoundrels. So far, pure effects of p1 (elites) and p3 (scoundrels) are ultimately figured out via the asymmetric model.

**Table 2:** Conditional Effects of Elites and Scoundrels.

	b = 1.1	b = 1.2	b = 1.3	b = 1.4	b = 1.5	b = 1.6	b = 1.7	b = 1.8	b = 1.9	b = 2.0
β_1_(·|b)	.66*** ^a^ (.0028)	.80*** (.0035)	.87*** (.0045)	.91*** (.0053)	.94*** (.0063)	.95*** (.0071)	.96*** (.0076)	.97*** (.0080)	.97*** (.0089)	.97*** (.0114)
β_3_(·|b)	-.73*** (.0028)	-.57*** (.0035)	-.45*** (.0045)	-.37*** (.0053)	-.30*** (.0063)	-.25*** (.0071)	-.22*** (.0076)	-.19*** (.0080)	-.16*** (.0089)	-.14*** (.0114)
Adj. *R* ^2^	0.9988^b^	0.9977	0.9959	0.9940	0.9919	0.9900	0.9887	0.9880	0.9858	0.9868

Table 1 gives OLS regression for Eq (3) conditioned at each level of b, from 1.1 to 2.0. As b changes, conditional effects of elites and scoundrels, β_1_(·|b) and β_1_(·|b), vary accordingly, and they are all statistically significant at the 0.001 level. Ten columns or conditional models fit well as the percentages of explained variance are beyond 98% and close to 100%.

^a^. Significance Level:

*** p < 0.001 (two-tailed)

^b^. Conditional models fit well for all adjusted R^2^ is above 0.98.

### Temptation’s Threshold

**Fig 8:**
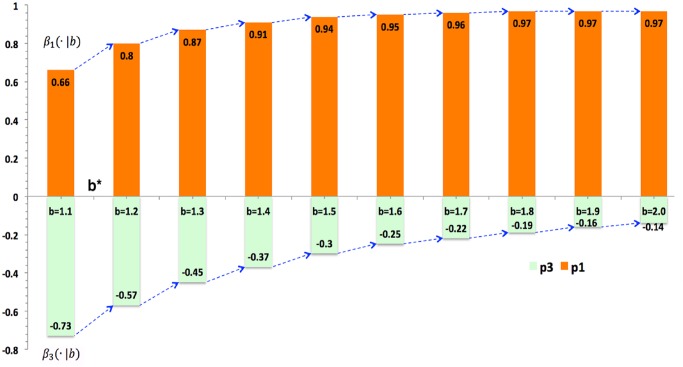
Conditional Effects of Elites and Scoundrels. As the temptation effect is regular, we control it to investigate effects of elites and scoundrels and it shows that elites promotes cooperation while scoundrels undermine cooperation or cultivate defection all the time. As b grows from 1.1 to 2.0, elites (p1) are playing a more and more positive role, as its coefficient *β*_1_(·|*b*) increases with b. Meanwhile, the negative effect of scoundrels’ *β*_3_(·|*b*) is getting weaker and weaker as temptation grows. The threshold b* is between 1.1 and 1.2, for elites’ effect is bigger than that of scoundrels as b > b* and weaker than it when b < b*.

It indicates in Table 2 that coefficients vary with temptation. Fig 7 visualizes coefficients of elites and scoundrels so that directions and magnitudes of their pure effects can be easily perceived. The symmetric model has shown that the more stratification brings higher cooperation rate when b > b*. This is mainly because elites’ positive effect conquers scoundrels’ negative effect. As Fig 8 tell us, when b is larger than its threshold b* that is within [1.1, 1.2], elites conquer scoundrels, which plays a supportive role in promoting cooperation. When b < b*, elites cannot countervail scoundrels, which is bad for cooperation. Therefore, elites plays a more and more important role while the scoundrel exerts less and less negative influence on cooperation as temptation increases.

**Fig 9:**
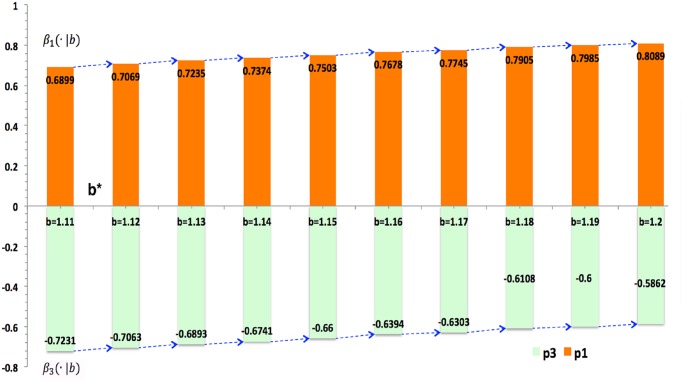
Conditional Effects of Elites and Scoundrels (Denser). The threshold b* is within 1.11 and 1.12 after b takes denser values within the interval [1.1, 1.2]. When b > b*, elites’ positive effect conquers scoundrels’ negative effect on the overall cooperation. However, it cannot overcome the bad effect when b < b*.

Hence, there ought the threshold of temptation, where effects of elites and scoundrels are equal and cancel out each other, i.e. they counteract each other and the overall effect is zero. Scoundrels win over elites while b < b* because *β*_1_(·|*b* < b*) < *β*_3_(·|*b* < b*), and elites defeat scoundrels when b > b* because *β*_1_(·|*b* < b*) < *β*_3_(·|*b* < b*). Fig 8 shows that b* is within the interval [1.1, 1.2]. In order to get more accurate evaluations of b*, this interval is zoomed in and b takes on values from the set {1.11, 1.12, 1.13… 1.19, 1.20}. It indicates in Fig 9 that b* ∈ (1.11, 1.12). For cooperation clusters, agents cooperate with each other. However, boundary agents of cooperating clusters have to cooperate with defectors. In order to remain the cooperating of them, there ought to be a temptation level that stabilizes boundary cooperators to have no reason to defect and remain cooperation. It is believed that this stable temptation is the threshold b*.

### Conditional Stratification Equations

**Fig 10:**
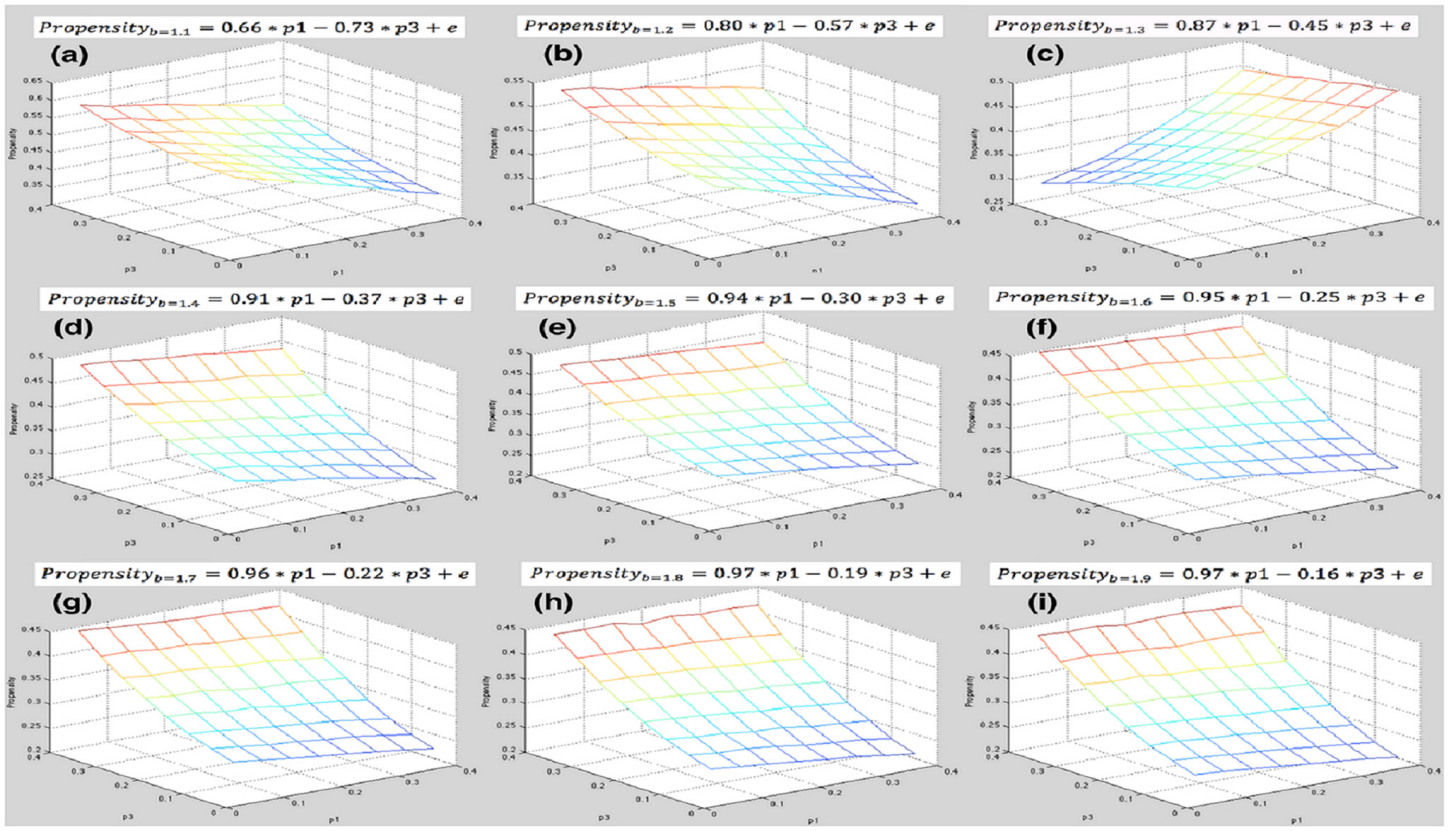
Conditional Stratification Equations. Elites’ and scoundrels’ effects vary on the temptation level. Based on coefficients of p1 and p3 in Table 2, stratification equations are obtained based on levels of b. (a) depicts the relationship of p1, p3, and *p*_*c*_ when b = 1.1, (b) depicts this relationship as b = 1.2, and so on. For each subgroup, the equation is listed as the subtitle.

Table 2 makes it possible to evaluate or predict effects of elites and scoundrels based on b, and the confident level is over 97%. Hence, conditional stratification equations consequently are obtained from it in Fig 10. There exist unique equations according to different values of b. From the perspective of 3-D plot in Fig 10, it can be seen that slopes of p1 and p3 are different when b varies. For each level of b, influences of elites and scoundrels are different. As b gets larger and larger, the slope of p1 increases and the absolute value of p3’s slope decreases at the same time, which means that elites are playing a more and more critical role while scoundrels’ negative effects are getting weaker and weaker as b increases.

## Discussions and Conclusions

The cooperation level and individual choice are influenced by mechanisms of stratification and temptation. In terms of stratification, two classes, elites and scoundrels are derived from the commons, and these three classes form the whole society. Symmetric models are used to preliminarily check out effects of stratification and temptation effect, and it indicates again that temptation reduces cooperation permanently [1, 4, 6]. Asymmetric models are utilized to evaluate pure effects of three factors such as b, p1, and p3. Statistical outcomes show that b and p3 play negative roles in maintaining cooperation. And this is caused by two reasons: temptation seduces agents of all classes to defect, and the other is that the scoundrel defects naturally. However, hope still exists for elites play a positive role in promoting cooperation. There are also two reasons for that: elites tend to cooperate naturally, and elites influence or encourage commons to cooperate rather than defect.

The stratification mechanism is conditioned on temptation and there seems to be a threshold of temptation. Effects of elites and scoundrels differ significantly as b varies. Conditioned on b, pure effects of elites and scoundrels can be extracted. It shows that as b increases, elites’ positive effect is getting stronger and stronger while scoundrels’ negative effect getting weaker and weaker. This means that there ought to be a threshold of temptation b*, where pure effects of elites and scoundrels balance out each other, i.e. the pure effect of scoundrels conquers that of elites when b is smaller, and the pure effect of elites conquers that of scoundrels when b grows larger. Only when b is larger than b* can elites class overcome negative effects of scoundrels, in that the boundary payoff of elites is larger than that of scoundrels as b>b*. Likewise, as b<b*, the boundary payoff of scoundrels is larger than that of elites and scoundrels win over elites and cooperation is therefore reduced. In order to evaluate this threshold, statistical models and methods are applied, and b* is found to be within the interval of [1.11, 1.12]. Based on different values of b, it is feasible to solve stratification equations and plot them. In all, elites play a more and more important role when temptation grows, and the stratification has a higher level of cooperation when b surpasses its threshold.

## Supporting Information

S1 Dataset The dataset of Fig 4. (XLSX)

S2 Dataset The dataset of Fig 5. (XLSX)

S3 Dataset The dataset of Fig 6. (XLSX)

S4 Dataset The dataset of Fig 7. (XLSX)

S5 Dataset The dataset of Fig 8. (XLSX)

S6 Dataset The dataset of Fig 9. (XLSX)

S7 Dataset The dataset of Fig 10. (XLSX)
